# Glutathione S-Transferase Polymorphisms (GSTM1, GSTT1 and GSTP1) and Their Susceptibility to Renal Cell Carcinoma: An Evidence-Based Meta-Analysis

**DOI:** 10.1371/journal.pone.0063827

**Published:** 2013-05-22

**Authors:** Xingliang Yang, Shuyu Long, Jianping Deng, Tianxing Deng, Zhihua Gong, Ping Hao

**Affiliations:** 1 Department of Urology, Xinqiao Hospital, Third Military Medical University, Chongqing, China; 2 Department of Gynecology and Obstetrics, West China Second Hospital, Sichuan University, Chengdu, Sichuan, China; 3 Department of Oncology, Xinqiao Hospital, Third Military Medical University, Chongqing, China; IPO, Inst Port Oncology, Portugal

## Abstract

**Background:**

The association of the three Glutathione S-transferases (GSTs) polymorphisms (GSTM1, GSTT1 and GSTP1) genotypes with their individual susceptibilities to renal cell carcinoma (RCC) has not been well established. We performed a quantitative meta-analysis to assess the possible associations between the GSTM1, GSTT1 and GSTP1 genotypes and their individual susceptibilities to renal cell carcinoma.

**Methods:**

We systematically searched the PubMed, CNKI and Embase databases to identify the relevant studies. Finally, 11 eligible studies were selected. The pooled odds ratios (ORs) with their 95% confidence intervals (CIs) were used to assess the association between the GSTs polymorphisms and the risk of RCC. Multiple subgroup analyses and quality assessment of the included studies were performed based on the available information.

**Results:**

None of the GSTs polymorphisms had a significant association with the RCC risk. Similar results were found in the subgroup analyses, except for the GSTs polymorphisms in the situations described below. The GSTM1 and GSTT1 active genotypes in subjects exposed to pesticides (GSTM1: OR = 3.44; 95% CI, 2.04–5.80; GSTT1: OR = 2.84; 95% CI, 1.75–4.60), most of the GSTs genotypes in Asian populations (GSTT1: OR = 2.39, 95% CI = 1.63–3.51; GSTP1: Dominant model: OR = 1.50, 95% CI = 1.14–1.99; Additive model: OR = 1.39, 95% CI = 1.12–1.73; AG *vs.* AA: OR = 1.47, 95% CI = 1.10–1.97; GG *vs.* AA: OR = 1.82, 95% CI = 1.07–3.09) and the dual null genotype of GSTT1-GSTP1 (OR = 2.84, 95% CI = 1.75–4.60) showed positive associations with the RCC risk.

**Conclusion:**

Our present study provides evidence that the GSTM1, GSTT1 and GSTP1 polymorphisms are not associated with the development of RCC. However, more case-control studies are needed for further confirmation.

## Introduction

In 2008, approximately 271,000 cases of kidney cancer were diagnosed around the world, and 116,000 individuals died of kidney cancer [1]. Renal cell carcinoma (RCC) accounts for the majority of kidney cancers (80–85%) and is the third most commonly diagnosed genitourinary malignancy [2]. Globally, the incidence of RCC varies by more than 10-fold between populations and geographic areas and has been rising steadily each year during the last three decades in Europe and the United States [3]–[5]. Despite the increasing incidence and considerable researches on RCC, its causes are not yet fully understood. Evidence suggests that smoking, obesity, hypertension and occupational exposure to chemicals are the important factors that contribute to the tumorigenesis of RCC [6]–[8]. However, RCC only develops in a small group of people who are exposed to the above factors, which suggests that genetic host factors might contribute to the carcinogenic mechanisms. Moreover, the evidence indicates that the development of RCC can be partially explained by genetic variations among the populations.

Glutathione S-transferases (GSTs) are a large family of Phase II detoxification enzymes that are expressed in many tissues and play critical roles in regulating the conversion of toxic compounds to hydrophilic metabolites [9]–[10]. Because the differential expression of GSTs has been found to markedly influence the anticarcinogenic potential of tissues since it was first suggested as a potential marker for cancer susceptibility in 1986 [11], GSTs are currently being investigated as risk biomarkers for various cancers, including RCC [12]–[15]. Among the GSTs, the association of the GSTM1, GSTT1 and GSTP1 genotypes with their individual susceptibilities to cancer has been extensively studied. GSTM1 is located on the short arm of chromosome 1 (1p13.3) [16], whereas GSTT1 is located on the long arm of chromosome 22 (22q11.23) [17]. Both genes have a null variant allele, which results in an absence of enzyme activity. Individuals who carry homozygous deletions in these genes are thought to be increased risks for malignancies because of their decreased capacity to detoxify potential carcinogens [18], [19]. The GSTP1 gene is located on chromosome 11 [18], and the single nucleotide polymorphisms (SNPs) in this gene are known to cause genetic damage and increased cancer risk [20]. The most common mutation is an A-to-G transition in codon 105 (rs1695, A105G), which results in an amino acid substitution of valine for isoleucine [21], [22].

Several studies were designed to evaluate the associations between these three GSTs genotypes and the susceptibility to RCC [23]–[33]; however, the results were inconsistent. The majority of the case-control genetic studies revealed no association between RCC and GSTs SNPs [23]–[29]. Some evidence indicated that the GSTs variants are positively associated with RCC risk [30], [31], whereas other evidence indicated that the GSTs variants are inversely associated with RCC risk [32], [33]. These inconclusive results may be due to the limited sample size which may be too underpowered to detect the precise effects. In addition, there may also be differences in the study characteristics, such as ethnicity, pathological history, sources of controls, and source of DNA for genotyping. With respect to GSTM1, GSTT1 and GSTP1, there is still a lack of firm evidence regarding the association between these three GSTs polymorphisms and RCC risk based on a quantitative analysis. Consequently, we performed this meta-analysis by combining the data from case-control studies to provide strong evidence for the association between GSTs polymorphisms and susceptibility to RCC.

## Materials and Methods

### Literature Search Strategy and Inclusion Criteria

We systematically searched the PubMed, CNKI (Chinese National Knowledge Infrastructure) and Embase databases (the last search was performed on December 17, 2012) using the keywords (“polymorphism” or “SNPs” or “Single Nucleotide Polymorphism”) and (“GST” or “glutathione S-transferase” or “GSTM1” or “glutathione S-transferase M1” or “GSTT1” or “glutathione S-transferase T1” or “GSTP1” or “Glutathione S-Transferase pi”) and (“RCC” or “Renal cell carcinoma” or “Renal Cell Cancer” or “Nephroid Carcinoma” or “Carcinoma, Renal Cell” or “Sarcomatoid Renal Cell Carcinoma” or “Clear Cell Renal Cell Carcinoma” or “Chromophobe Renal Cell Carcinoma” or “Chromophil Renal Cell Carcinoma” or “Adenocarcinoma, Renal Cell”) without language restriction to identify the relevant studies. Reference lists of the identified articles were also examined, and the literature retrieval was conducted in duplicate by two independent reviewers (XY and SL).

Studies concerning the association of GSTM1, GSTT1 and/or GSTP1 polymorphisms with RCC susceptibility were included. The eligible studies had to meet the following inclusion criteria: (1) the studies should assess the relationship between GSTM1, GSTT1 and/or GSTP1 polymorphism(s) and RCC susceptibility; (2) the studies should be case–control studies; and (3) the studies should provide sufficient data for inferring odds ratios (ORs) and their corresponding 95% confidence intervals (95% CIs). The exclusion criteria were as follows: (1) a review, case report, editorial, or comment; (2) a duplicate study; (3) laboratory molecular or animal studies; (4) when multiple studies reported the same data, the most recent studies or those with the largest sample sizes were selected, and the other studies were excluded.

Because the data included in this study were taken from the literature, written consent from the patients and ethical approval from ethics committees were not needed.

### Data Extraction

The data from the eligible studies that were selected in strict accordance with the inclusion criteria were independently extracted by two investigators (XY and SL). The controversial issues were resolved after discussion. The following data were extracted from each study: the first author’s name, the year of publication, the country, the ethnicity, the source of controls, the goodness-of-fit of Hardy-Weinberg Equilibrium (HWE) in the control group, the total number of cases and controls with various genotypes, and the distribution of the respective genotypes in the case and control groups. With respect to the studies that provided inadequate information, the authors were contacted by e-mail for further information if possible.

### Quality Score Assessment

The quality of the included studies was independently assessed by two investigators (XY and SL) using the quality assessment criteria, which were amended compared to those used in the previously published meta-analytic studies [34], [35]. The following factors were included in the criteria (Table S1): representativeness of the case, representativeness of the control, determination of renal cell carcinoma, genotyping examination, matching of case and control participants, and total sample size. Each component was evaluated on a scale from 0 to 12. If the score was ≥7, the study was categorized as “high quality”; otherwise, the study was categorized as “low quality. All disagreements were resolved by consensus after discussion.

### Statistical Analysis

Statistical analysis was performed with the RevMan 5.0 program (Cochrane Collaboration) and the STATA package version 11.0 program (Stata Corporation, College Station, TX). The ORs and the corresponding 95% CIs were used to estimate the strength of the associations between the GSTM1, GSTT1 and GSTP1 polymorphisms and RCC risk. Because there were only two genotypes (null and active) for the GSTM1 and GSTT1 genes, the pooled ORs were performed only between these two genotypes. However, there were three genotypes (AA, AG and GG) for the GSTP1 gene; therefore, the pooled ORs were performed for the dominant model (GG+AG vs. AA), recessive model (GG vs. AG+AA) and additive model (A vs. G). Moreover, the pooled estimates were also calculated for the pair-wise comparisons (allele AG vs. AA, and allele GG vs. AA). The chi-square-based Q-test and I^2^ statistics were used to assess the statistical heterogeneity among the studies [36], [37]. When a statistically significant result was obtained from a Q test (*P*<0.10 or I^2^>50%), heterogeneity was considered to exist across studies, and the random-effects model was used to calculate the pooled OR; however, in other cases, the fixed-effects model was used. Subgroup analysis, which was used to explore and explain the heterogeneity between the different studies, was performed based on the combined effects of the three genotypes, geographic area, source of controls, quality score of the included studies and occupational exposure. If the genotype data were available, the Hardy–Weinberg equilibrium (HWE) was tested in the controls used for each study via the chi-square test. Sensitivity analyses were performed to assess the stability of the results: case-control studies were omitted from all the iterations to reflect the influence of the individual data set on the pooled OR. Asymmetry in the funnel plot indicated a possible publication bias. In addition, the Egger’s and Begg’s quantitative tests were also used, and *P*<0.05 was considered as statistically significant [38], [39]. To ensure the reliability of the data, two reviewers (XY and SL) independently performed the data analysis using the statistical programs to manipulate the same data.

## Results

### Selection and Characteristics of the Studies

**Figure 1:**
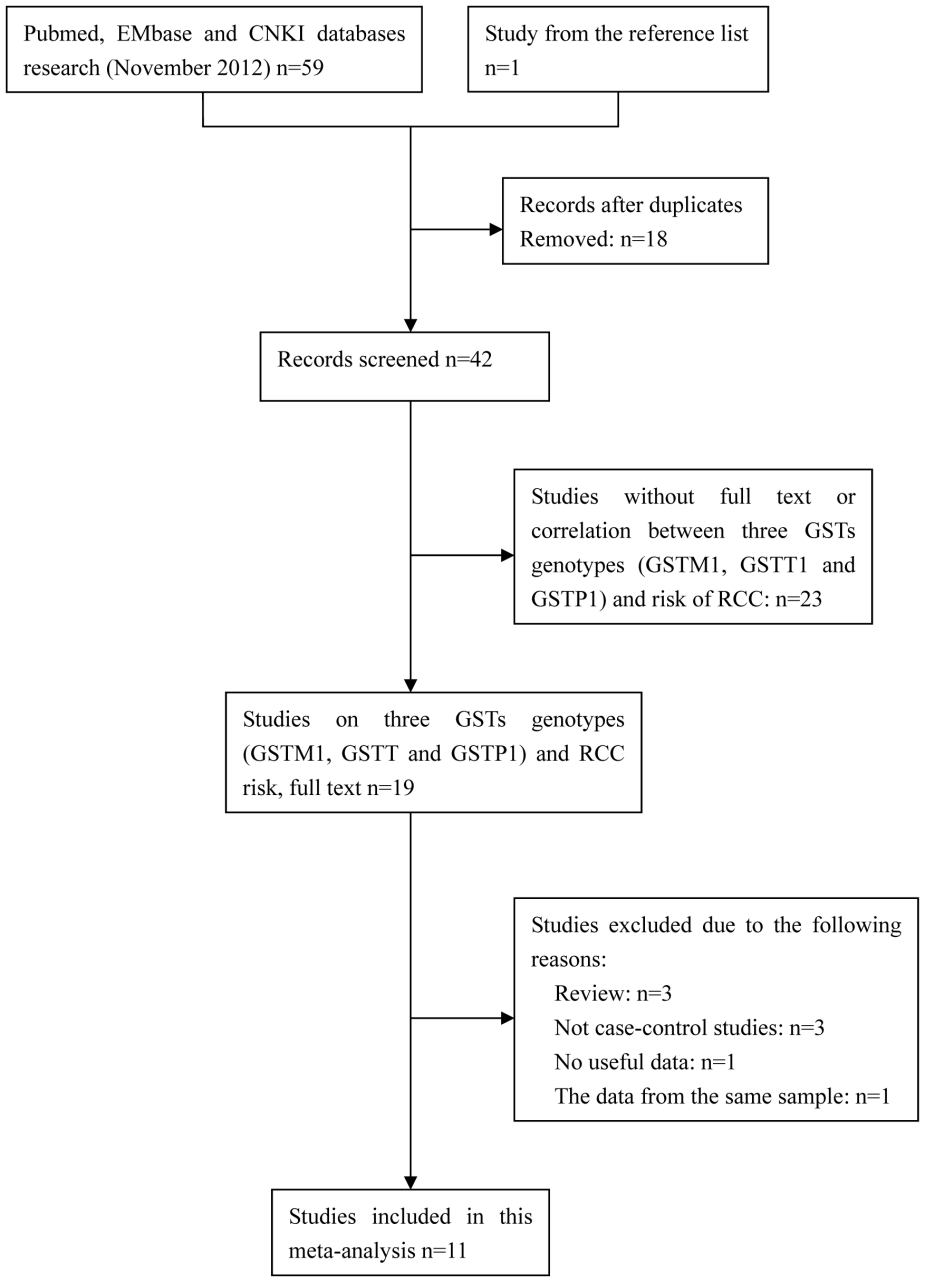
Flow diagram of the selection process for the eligible studies.

After performing a careful search, 60 potentially relevant publications were identified. Based on the inclusion criteria, 11 studies (10 for GSTM1 and GSTT1, 5 for GSTP1) [23]–[33] were eligible for this meta-analysis. A flow chart describing the selection process for the eligible studies is shown in Figure 1. During the selection process, 49 studies were removed: 18 studies were duplicate studies; 23 studies were not available in full text or did not show a correlation between the three GSTs genotype (GSTM1, GSTT1 and GSTP1) polymorphisms and risk of RCC; 3 studies were reviews; 3 studies were not case-control studies; 1 did not provide any useful data; and 1 studie provided the same data as another included one. Finally, 1718 cases and 2912 controls were included for the analysis of the GSTM1 genotype, 1721 cases and 2907 controls were used for the analysis of the GSTT1 genotype and 792 cases and 1491 controls were included for the analysis of the GSTP1 genotype. The main characteristics of the included studies in terms of the different genotypes are summarized in Table 1 and 2 respectively. The HWE was tested in each study. The 5 studies for the GSTP1 gene showed results that were not significant (NS) and conformed to Hardy-Weinberg Equilibrium (HWE) expectations (*P*>0.05). However, for the GSTM1 and GSTT1 genes, the HWE test was conducted in only one [23] article, and the conditions met the Hardy-Weinberg equilibrium. Because only two genotypes (null and active) were included in the remaining studies, the HWE could not be evaluated. With respect to the assessment of the quality of the included studies, as shown in Tables 1–3, 9 articles were identified as “high quality” [24]–[32], and 2 articles were identified as “low quality” [23], [33].

**Table 1:** Characteristics of the included case-control studies in the meta-analysis of the association between GSTM1 and GSTT1 polymorphisms and the risk of renal cell carcinoma*.

First author	Year	Country	Ethnicity	Source ofcontrols	Quality	GSTM1			GSTT1		
[reference]					Score	Sample size	Case	Control	Sample size	Case	Control
						case/control	null/active	null/active	case/control	null/active	null/active
Salinas-Sánchez [25]	2010	Spain	Caucasian	Hospital-based	6	133/193	57/76	78/115	132/163	22/110	25/138
Wiesenhütter [23]	2007	Germany	Caucasian	Hospital-based	8	98/324	51/47	167/157	98/324	19/79	59/265
Karami [26]	2008	Central and Eastern Europe	Caucasian	Hospital-based	9	624/887	303/321	433/454	628/913	129/499	161/752
Buzio [29]	2003	Italy	Caucasian	Hospital-based	8	100/200	50/50	108/92	100/200	11/89	35/165
Sweeney [30]	2000	USA	Mixed	Population-based	9	126/505	63/63	255/250	126/504	36/90	93/411
De Martino [28]	2010	Austria	Caucasian	Hospital-based	8	147/112	80/67	59/53	147/112	27/120	23/89
Longuemaux [31]	1999	France	Caucasian	Hospital-based	8	173/211	89/84	117/94	173/211	25/148	40/171
Ahmad [24]	2012	India	Asian	Population-based	11	196/250	102/94	116/134	196/250	125/71	106/144
Ćorić [27]	2010	Serbia	Caucasian	Hospital-based	8	76/182	46/30	86/96	76/182	21/55	52/130
Bruning [33]	1997	Germany	Caucasian	Population-based	6	45/48	18/27	31/17	45/48	3/42	11/37

* The null genotype: no active allele; the active genotype: more than one active allele.

**Table 2:** Characteristics of the included case-control studies in the meta-analysis of the association between GSTP1 polymorphism and the risk of renal cell carcinoma.

First author	Year	Country	Ethnicity	Source of	HWE	Quality	Sample size		Case						Control					
[reference]				controls		Score	Case	Control	A	G	AA	AG	GG	AG+GG	A	G	AA	AG	GG	AG+GG
Ahmad [24]	2012	India	Asian	Population-based	NS	11	196	250	241	151	71	99	26	125	355	145	126	103	21	124
Wiesenhütter [23]	2007	Germany	Caucasian	Hospital-based	NS	8	99	325	141	57	49	43	7	50	412	238	134	144	47	191
Sweeney [30]	2000	USA	Mixed	Population-based	NS	9	130	491	172	88	58	56	16	72	642	340	213	216	62	278
Longuemaux [31]	1999	France	Caucasian	Hospital-based	NS	8	160	189	209	111	71	67	22	89	261	117	93	75	21	96
Wang [32]	2011	China	Asian	Hospital-based	NS	9	207	236	341	73	143	55	9	64	400	72	173	54	9	63

Abbreviations: HWE, Hardy-Weinberg Equilibrium; NS, not significant.

**Table 3:** Pooled Analysis of the Association between GSTM1 polymorphism and RCC risk.

Genetic model	Number ofstudies	Sample Size		Analysis	I^2^ (%)	*P*_h_	Test of Association		*P* (Publication bias test)	
(GSTM1)		Case	Control	Model			*P*	OR(95% CI)	Begg’s test	Egger’s test
Total	10	1718	2912	F	25	0.22	0.85	1.01 [0.90, 1.14]	1.000	0.949
Source of controls										
Hospital-based	7	1351	2109	F	0	0.54	0.83	1.02 [0.88, 1.17]		
Population-based	3	367	803	R	71	0.03	0.61	0.87 [0.51, 1.47]		
Geographic area										
European	8	1396	2157	F	34	0.16	0.86	0.99 [0.86, 1.13]		
Asian	1	196	250				0.24	1.25 [0.86, 1.82]		
USA	1	126	505				0.91	0.98 [0.66, 1.45]		
Quality Score										
High quality	8	1540	2671	F	0	0.53	0.65	1.03 [0.91, 1.17]		
Low quality	2	178	241	R	81	0.02	0.47	0.67 [0.23, 1.98]		
Occupational exposure										
active+ exposure	3	387	580	F	0	0.44	<0.01	3.44 [2.04, 5.80]		
null+ exposure	3	381	601	F	0	0.91	0.04	1.62 [1.02, 2.57]		

Abbreviations: R, Random-effects model; F, Fixed-effects model; P_h,_ test for heterogeneity.

### Quantitative Synthesis

#### Association between GSTM1 Polymorphisms and RCC risk

**Figure 2:**
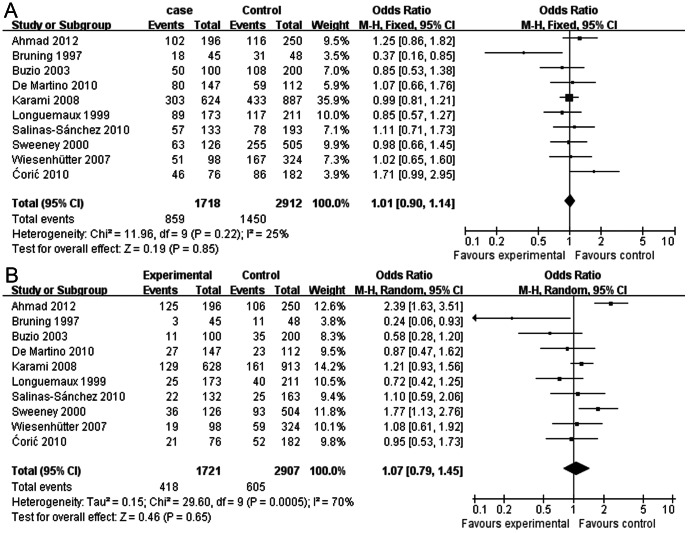
Meta-analysis of the association between GSTM1/GSTT1 polymorphisms and RCC risk. (A) Meta-analysis of the association between GSTM1 polymorphism and RCC risk using the fixed-effects model. (B) Meta-analysis of the association between GSTT1 polymorphism and RCC risk using a random-effects model. The area of the squares reflects the weight (inverse of the variance). The diamond represents the summary OR and 95% CI.

No association was found between the GSTM1 null polymorphism and the susceptibility to RCC in the overall population (Table 3, *P* = 0.85, OR = 1.01, 95% CI = 0.90–1.14) (Figure 2A). The heterogeneity in the GSTM1 genetic models was not significant (*P* = 0.22, I^2^ = 25%), and the fixed-effects model was selected to calculate the pooled results in the meta-analysis. Although multiple subgroup analyses of geographic area, source of controls and quality scores revealed no association between the GSTM1 polymorphisms and RCC risk, the subgroup analysis of occupational exposure to pesticides showed a positive association between the GSTM1 polymorphisms and RCC risk (active+ exposure: *P*<0.05, OR = 3.44, 95% CI = 2.04–5.80; null+ exposure: *P* = 0.04, OR = 1.63, 95% CI = 1.02–2.57).

#### Association between GSTT1 Polymorphisms and RCC risk

No association was found between the GSTT1 null polymorphism and the susceptibility to RCC in the overall population (Table 4, P = 0.65, OR = 1.07, 95% CI = 0.79–1.45) (Figure 2B). However, heterogeneity was identified (*P*<0.05, I^2^ = 70%) in the GSTT1 genetic models; therefore, the random-effects model was used for the analysis. As for the subgroup analysis of populations in different geographic areas, the heterogeneity was significantly decreased after the classification (*P* = 0.18, I^2^ = 32%). The pooled data in the fixed-effects model used for the European subgroup (*P* = 0.89, OR = 0.99, 95% CI = 0.83–1.18) indicated that the GSTT1 null genotype in European populations had no significant association with the RCC risk. Similar results were obtained from the high-quality studies in European populations. Interestingly, the Asian and US populations showed positive associations between the GSTT1 polymorphisms and the susceptibility to RCC (Asian population: *P*<0.01, OR = 2.39, 95% CI = 1.63–3.51; US population: *P* = 0.01, OR = 1.77, 95% CI = 1.13–2.76). As for subgroup analysis according to the source of control and quality score, no association was found between the GSTT1 null polymorphism and RCC risk. In addition, positive results were found in the subgroup analysis of occupational exposure to pesticides (active+ exposure: *P*<0.01, OR = 2.58, 95% CI = 1.57–4.60).

**Table 4:** Pooled Analysis of the Association between GSTT1 polymorphism and RCC risk.

Genetic model	Number ofstudies	Sample Size		Analysis	I^2^ (%)	*P*_h_	Test of Association		*P* (Publication bias test)	
(GSTT1)		Case	Control	Model			*P*	OR(95% CI)	Begg’s test	Egger’s test
Total	10	1721	2907	R	70	<0.05	0.65	1.07 [0.79, 1.45]	0.152	0.236
Source of controls										
Hospital-based	7	1354	2105	F	0	0.44	0.80	1.02 [0.86, 1.22]		
Population-based	3	367	802	R	81	<0.05	0.43	1.37 [0.63, 2.96]		
Geographic area										
European	8	1399	2153	F	32	0.18	0.89	0.99 [0.83, 1.18]		
European*	6	1222	1942	F	14	0.32	0.91	1.01 [0.84, 1.22]		
Asian	1	196	250				<0.01	2.39 [1.63, 3.51]		
USA	1	126	504				0.01	1.77 [1.13, 2.76]		
Quality Score										
High quality	8	1544	2696	R	71	<0.05	0.39	1.15 [0.84, 1.57]		
Low quality	2	177	211	R	75	0.04	0.47	0.58 [0.13, 2.56]		
Occupational exposure										
active+ exposure	3	209	279	F	0	0.50	<0.01	2.84 [1.75, 4.60]		
null+ exposure	3	159	240	F	34	0.22	0.06	1.96 [0.96, 3.99]		

Abbreviations: R, Random-effects model; F, Fixed-effects model; P_h,_ test for heterogeneity. European*: two studies [23], [32] were omitted because of low quality.

#### Association between GSTP1 Polymorphisms and RCC risk

**Figure 3:**
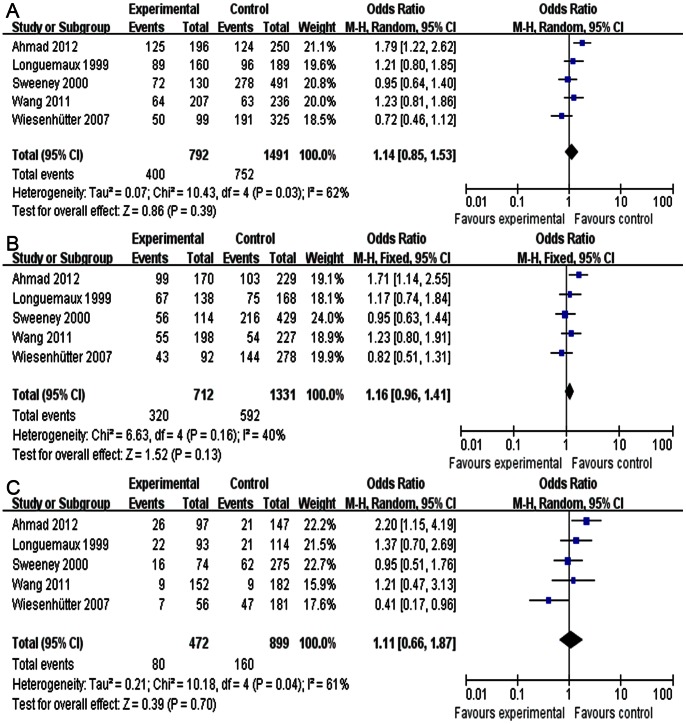
Meta-analysis of the association between GSTP1 polymorphisms and RCC risk. (A) Meta-analysis of AG+GG *vs.* AA (dominant model) using the random-effects model. (B) Meta-analysis of AG *vs*. AA using the fixed-effects model. (C) Meta-analysis of GG *vs.* AA using a random-effects model. The area of the squares reflects the weight (inverse of the variance). The diamond represents the summary OR and 95% CI.

No association was found between the GSTP1 polymorphisms and the susceptibility to RCC in all the genetic models (Table 5, dominant model: OR = 1.14, 95% CI = 0.85–1.53, Figure 3A; recessive model: OR = 1.05, 95% CI = 0.78–1.42; additive model: OR = 1.09, 95% CI = 0.84–1.40; AG *vs.* AA: OR = 1.16, 95% CI = 0.96–1.41, Figure 3B; GG *vs.* AA: OR = 1.11, 95% CI = 0.66– 1.87, Figure 3C). The heterogeneity was significant in most of the genetic models (*P*<0.05), and the random-effects model was used for the meta-analysis. As for the subgroup analysis of populations in different geographic area, the heterogeneity was dramatically decreased in Asian populations (*P*>0.05), and a positive association was found between the GSTP1 polymorphisms and the susceptibility to RCC in most of the genetic models (dominant model: *P*<0.01, OR = 1.50, 95% CI = 1.14–1.99; additive model: *P*<0.01, OR = 1.39, 95% CI = 1.12–1.73; AG *vs.* AA: *P* = 0.01, OR = 1.47, 95% CI = 1.10–1.97; GG *vs.* AA: *P* = 0.03, OR = 1.82, 95% CI = 1.07– 3.09). Subgroup analysis of the source of controls was also performed; however, no decrease in the heterogeneity across the studies was detected (data not shown). The subgroup analysis of quality score is not shown because all of the included studies were high quality.

**Table 5:** Pooled Analysis of the Association between GSTP1 polymorphism and RCC risk.

Genetic model	Number ofstudies	Sample Size		Analysis	I^2^ (%)	*P*_h_	Test of Association		P(Publication bias test)	
		Case	Control	Model			*P*	OR (95% CI)	Begg’s test	Egger’s test
Total										
Dominant model	5	792	1491	R	62	0.03	0.39	1.14 [0.85, 1.53]	0.221	0.223
Recessive model	5	792	1491	F	40	0.15	0.73	1.05 [0.78, 1.42]	0.806	0.456
Additive model	5	1584	2982	R	69	0.01	0.52	1.09 [0.84, 1.40]	0.462	0.379
AG *vs.* AA	5	712	1331	F	40	0.16	0.13	1.16 [0.96, 1.41]	0.221	0.220
GG *vs.* AA	5	472	899	R	61	0.04	0.70	1.11 [0.66, 1.87]	0.806	0.488
Geographic area										
European										
Dominant model	2	259	514	R	64	0.09	0.81	0.94 [0.56, 1.57]		
Recessive model	2	259	514	R	74	0.05	0.64	0.78 [0.28, 2.18]		
Additive model	2	518	1028	R	79	0.03	0.74	0.91 [0.55, 1.53]		
AG vs. AA	2	230	446	F	14	0.28	0.93	0.99 [0.71, 1.36]		
GG vs. AA	2	149	295	R	79	0.03	0.67	0.77 [0.23, 2.54]		
Asian										
Dominant model	2	403	486	F	42	0.19	<0.01	1.50 [1.14, 1.99]		
Recessive model	2	403	486	F	0	0.51	0.12	1.49 [0.90, 2.49]		
Additive model	2	806	972	F	17	0.27	<0.01	1.39 [1.12, 1.73]		
AG vs. AA	2	368	456	F	14	0.28	0.01	1.47 [1.10, 1.97]		
GG vs. AA	2	249	329	F	4	0.31	0.03	1.82 [1.07, 3.09]		

Dominant model: AG+GG *vs.* AA; Recessive model: GG *vs.* AA+AG; Additive model: G *vs.* A. Abbreviations: R, Random-effects model; F, Fixed-effects model; P_h_, test for heterogeneity.

#### Association between the combined effects of GSTs (GSTM1, GSTT1 and GSTP1) Polymorphisms and RCC risk

No association was found between the combined effects of GSTs polymorphisms and the susceptibility to RCC in most of the genetic models (Table 6, GSTM1(−)/GSTT1(−): OR = 1.06, 95% CI = 0.62–1.81; GSTM1(−)/GSTP1(−): OR = 1.51, 95% CI = 0.78–2.92; GSTM1(−)/GSTT1(−)/GSTP1(−): OR = 2.58, 95% CI = 0.78–8.53). The heterogeneity was significant in all of the genetic models (*P*<0.05), and the random-effects model was used for the meta-analysis. Because of the limitation of original studies, we focused on the analysis of the GSTM1(−)/GSTT1(−) genetic model. Considering that several factors might influence the heterogeneity, subgroup analyses of geographic area and quality score were performed to assess the association between the combined effects of the null genotypes (GSTM1 and GSTT1) and RCC risk. No association was found between the combined effects of the null genotypes (GSTM1 and GSTT1) and the susceptibility to RCC in the quality score subgroup analysis. However, several positive outcomes were found in the subgroup analysis of different geographic area (European population: *P* = 0.02, OR = 0.79, 95% CI = 0.65–0.92; Asian population: *P*<0.01, OR = 2.70, 95% CI = 1.61–4.52). To confirm this outcome more precisely, the results of the high-quality studies in the European population were assessed in this subgroup, and similar results were obtained (*P* = 0.03, OR = 0.80, 95% CI = 0.66–0.98). Furthermore, with respect to the GSTT1(−)/GSTP1(−) genetic model, a significant association was found between the combined effects of the null polymorphisms (GSTT1 and GSTP1) and the susceptibility to RCC (*P*<0.01, OR = 2.79, 95% CI = 1.44–5.42).

**Table 6:** Pooled analysis of the combined effects of GSTT1, GSTM1, and GSTP1 genotypes and RCC risk^&^.

Genetic model	Number ofstudies	Sample Size		Analysis	I^2^ (%)	*P*_h_	Test of Association	
		Case	Control	Model			*P*	OR(95% CI)
GSTM1(−)/GSTT1(−)								
Total	6	1052	1573	R	81	<0.05	0.84	1.06 [0.62, 1.81]
Geographic area								
European	4	880	1187	F	37	0.19	0.02	0.79 [0.65, 0.96]
European*	2	723	1008	F	0	0.56	0.03	0.80 [0.66, 0.98]
Asian	1	111	136				<0.01	2.70 [1.61, 4.52]
USA	1	61	250				0.16	1.58 [0.83, 3.01]
Quality Score								
High quality	4	895	1394	R	86	<0.05	0.50	1.22 [0.68, 2.18]
Low quality	2	157	179	R	77	0.04	0.43	0.36 [0.03, 4.56]
GSTM1(−)/GSTP1(−)	2	166	377	R	64	0.10	0.23	1.51 [0.78, 2.92]
GSTT1(−)/GSTP1(−)	2	172	354	R	61	0.11	<0.01	2.79 [1.44, 5.42]
GSTM1(−)/GSTT1(−)/GSTP1(−)	2	94	189	R	74	0.05	0.12	2.58 [0.78, 8.53]

Abbreviations: R, Random-effects model; F, Fixed-effects model; P_h,_ test for heterogeneity. ^&^ GSTM1 (−): the null genotype; GSTT1 (−): the null genotype; GSTP1 (−): AG or GG; European*: two studies [23], [32] were omitted because of low quality.

#### Publication bias and sensitivity analysis

**Figure 4:**
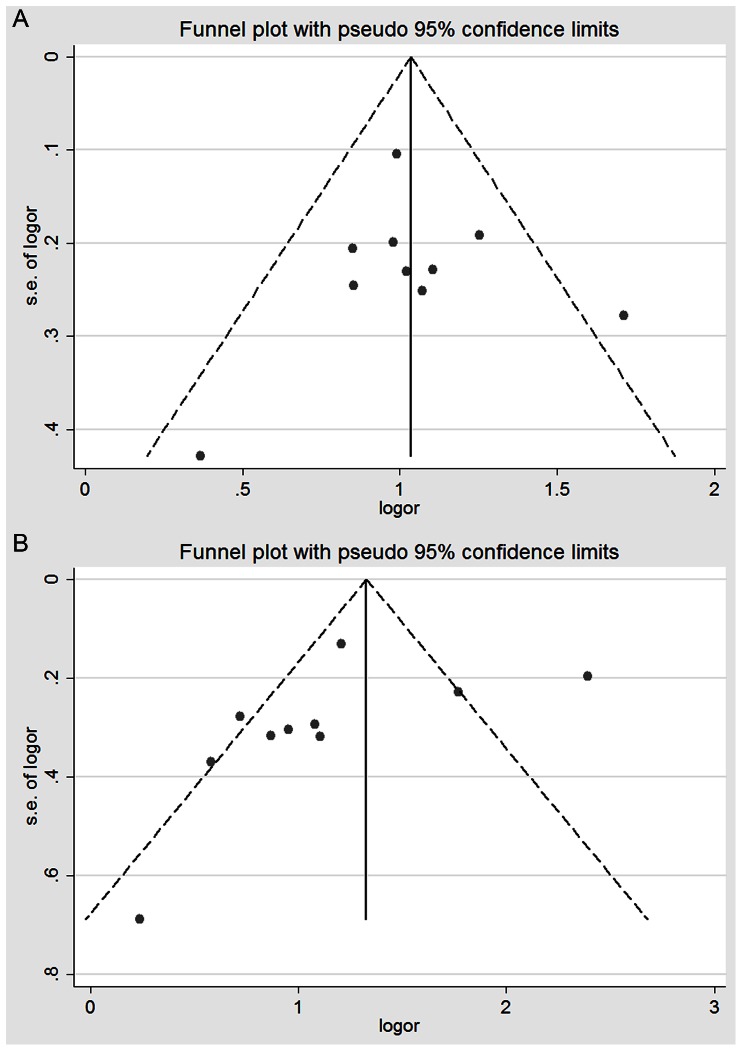
Funnel plot for the detection of the publication bias in the meta-analysis of the association between GSTM1/GSTT1 polymorphisms and RCC risk. (A) Funnel plot for the detection of bias in the meta-analysis of the association between GSTM1 null genotype and RCC risk using the fixed-effects model. (B) Funnel plot for the detection of bias in the meta-analysis of the association between GSTT1 null genotype and RCC risk using the random-effects model. Each point represents an individual study for the indicated association. LogOR, natural logarithm of OR. Perpendicular line denotes the mean effect size.

**Figure 5:**
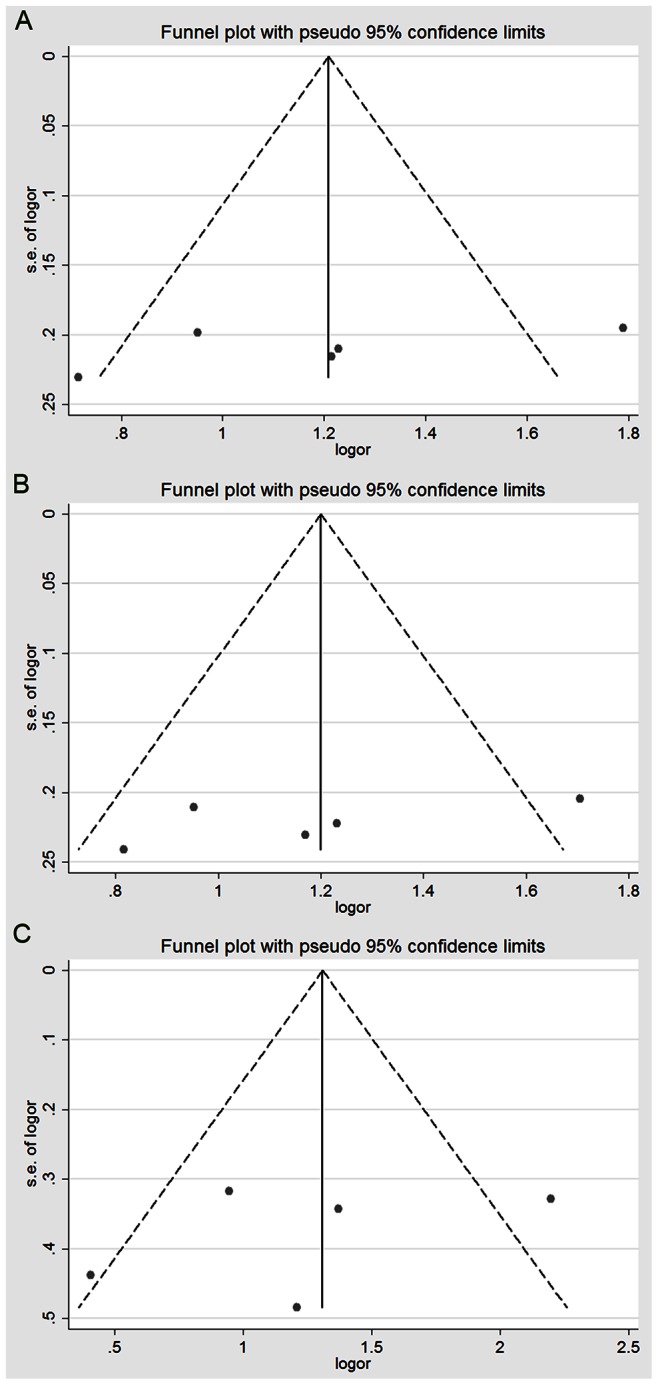
Funnel plot for the detection of the publication bias in the meta-analysis of the association between GSTP1 polymorphism and RCC risk. (A) Meta-analysis of AG+GG *vs*. AA (dominant model) using the random-effects model. (B) Meta-analysis of AG *vs.* AA using a fixed-effects model. (C) Meta-analysis of GG *vs.* AA using the random-effects model. Each point represents an individual study for the indicated association. LogOR, natural logarithm of OR. Perpendicular line denotes the mean effect size.

Publication bias was detected based in the shape of funnel plots and the Begg’s and Egger’s tests, as shown in Figure 4 and Figure 5. There was no obvious asymmetry in the charts. Similarly, there was no evidence of publication bias using the Begg’s and Egger’s tests, and the detailed general genotype data are summarized in Tables 1 and 2. Furthermore, there was no obvious evidence for the publication bias in the subgroup analysis in any of the genetic models that used the same methods (data not shown). Sensitivity analyses were conducted to assess the influence of each individual study on the pooled OR by removing one study at a time. In the overall meta-analysis, no single study changed the pooled results, which indicates that the results were statistically stable and reliable.

## Discussion

There are several studies of the relationship of GSTs polymorphisms and cancers. Among the members of the GST superfamily, GSTM1, GSTT1 and GSTP1 genotypes are considered to be the most related to the development of many cancers because their roles in lung cancer, acute leukemia and breast cancer have been identified in previous studies [12], [13], [40]. Similarly, there are several studies indicating a possible association between these genes and RCC risk; however, the results of these studies were not consistent. We found that none of these three GSTs polymorphisms had a significant association with the susceptibility to RCC. These results were consistent with a recent meta-analysis study conducted by Liu et al. [41] who analyzed the relevance between GSTM1 polymorphism and RCC risk. In addition, Cheng et al. [42] also demonstrated the association between the GSTM1 and GSTT1 polymorphisms and RCC risk in a meta-analysis, but there were some difference in conclusions between their studies and ours. Four papers [24], [27], [32], [33] included in our study showed a dramatic increase in the number of RCC cases and controls and provided available genetic information; however, this information was lacking in Cheng’s studies [42]. Furthermore, some data extracted from the included studies were controversial; the details are provided below. The number of GSTM1 null genotypes in the case and control groups extracted from the study by Longuemaux [28] and the number of GSTM1 null genotypes in the control group extracted from the study by Martino [24] were controversial. Similarly, the data of the GSTT1 genotype present in the control group extracted from the study by Karami [25] and those of the GSTT1 null genotype in the case group extracted from the study by Martino were controversial [28]. Therefore, to derive a more precise estimation of the relationship between the GSTs genotype (GSTM1, GSTT1 and GSTP1) polymorphisms and RCC risk, we performed this meta-analysis and included a larger number of studies. In addition, quality assessment of the included studies and multiple subgroup analyses which could sufficiently explore the heterogeneity were also performed in our meta-analysis. Most of the included studies [24]–[32] were high quality, except for two studies [23], [33]; this result indicates that the quality of all the included studies was high, which lends support to our conclusion. Furthermore, to the best of our knowledge, this study represents the first, large-scale meta-analysis of the association between the three GSTs genotype (GSTM1, GSTT1 and GSTP1) polymorphisms and susceptibility to RCC.

However, after performing a careful investigation according to a rigorous study design, we found that none of the three GSTs genotypes (GSTM1, GSTT1 and GSTP1) had a significant association with the risk of RCC. These results were consistent with most of the previous studies [23]–[29]. Although GSTs play critical roles in the development of tumors, in fact, it has been found that GSTs activity is lower in renal tumor tissue specimens than in healthy renal tissue distant to the tumor, and it’s also lower than in normal subjects [43]. Perhaps it did this reasons result that no association between GSTs polymorphisms and susceptibility to RCC were found. With respect to the GSTT1 genotype, the presence of heterogeneity among the included studies due to the geographic distribution forced us to exclude two studies [27], [30] in the Asian and US populations. No association was found between the GSTT1 polymorphism and RCC risk in the European populations. However, a significant positive association was found between the GSTT1 polymorphism and RCC risk in the Asian and US populations. A similar result was observed for the association between the GSTP1 genotype polymorphism and RCC risk in Asian populations. These data suggest that genetic background or environmental differences may contribute to the discrepancy in the results. However, in the present study, we cannot derive any conclusion because of the limited number of studies in the non-European populations; therefore, further studies are required. With respect to the exposure to pesticides, the occupationally exposed subjects with GSTM1 or GSTT1 active genotypes had a significantly increased risk for RCC compared with those of occupationally exposed subjects with GSTM1 or GSTT1 null genotypes and unexposed subjects. In general, glutathione compounds are excreted easily. However, in specific tissues, these compounds are more reactive than in normal tissues. These phenomena are especially evident in the kidneys [27], [44]. These more reactive intermediates damage the kidney tissues directly, and active GSTs enzymes are required for the formation of such intermediates. Conversely, the GSTs mutant genotype forms inactive enzymes and is responsible for the detoxification of carcinogens [27], [45]–[47]. This finding suggests that the subjects who are occupationally exposed to pesticides and have an active GSTM1 or GSTT1 variant have a significantly increased risk of RCC. Although a marginally positive effect was observed in the occupationally exposed subjects with a GSTM1 null genotype in statistics, based on the negative results of all original articles and the positive mechanism, this result may have no clinical significance.

Most of the genetic models showed no significant association between the combined effects of GSTs (GSTM1, GSTT1 and GSTP1) polymorphisms and RCC risk, except for the two dual null genotypes of GSTM1-GSTT1 and GSTT1-GSTP1. The findings for the dual null genotype of GSTM1-GSTT1 are interesting. A positive association was observed between the dual null genotype of GSTM1-GSTT1 and RCC risk in Asian populations; however, an inverse association was observed between the dual null genotype of GSTM1-GSTT1 and RCC risk in European populations, and we confirmed this result in the high-quality studies among European populations. Based on the lower heterogeneity among these studies, the pooled results could be considered to be reliable; thus, the discrepancy in the results could be explained by the differences in the ethnic backgrounds.

Similar to the other meta-analyses, this study has several limitations that need to be addressed. First, the overall sample size was not large enough. We need to perform more original studies to enhance the reliability and accuracy of our conclusions. In addition, the majority of the subjects included in the studies were Caucasian, and only two studies were conducted in Asian populations. Importantly, this study found interesting results, and an association was observed between the null genotypes and increased RCC risk. Therefore, to explain the discrepancy in the results caused by the different races of the subjects, more studies in other ethnic groups are needed. Second, some important information was unavailable and was not reported in the included studies, e.g., pathological subtypes, smoking status, BMI, hypertension or occupational exposure factors (such as solvents, metals and trichloroethylene). Therefore, the effects of pathological status, environmental exposure or lifestyle on the association between GST variants and RCC could not be determined in this meta-analysis. Third, because there are only two genotypes (null and active) at the GSTM1 and GSTT1 loci, the lack of data did not allow for the assessment of HWE.

In summary, despite the above-mentioned limitations, the present study provides evidence that the GSTM1, GSTT1 and GSTP1 polymorphisms are not associated with the development of RCC in the overall analysis. However, some specific associations between the GSTs polymorphisms and the susceptibility for RCC based on partial of the combined effects of GST genotypes, geographic area and occupational exposure to pesticides in the subgroup analysis were found. More case-control studies are needed to further demonstrate the association identified in the current meta-analysis.

## Supporting Information

Table S1 **Scale for Quality Assessment.**
(DOC)

Table S2 **PRISMA Checklist.**
(DOC)
